# Enterovirus 71 seropositivity among children and adolescents in Bangladesh

**DOI:** 10.1016/j.ijregi.2025.100739

**Published:** 2025-08-23

**Authors:** Sabeena Ahmed, Mohammad M. Karim, Ching S. Phru, Kamrun Nahar, Amina T. Sharmeen, Mohammad S. Hossain, Pappu C. Das, Mohammad A. Islam, Nusrat Jahan, Khondoker M. Jamil, Allen G. Ross, Muhammad J.A. Shiddiky, Wasif A. Khan

**Affiliations:** 1 International Centre for Diarrhoeal Disease Research, Bangladesh (icddr,b), Dhaka, Bangladesh; 2 Institute of Public Health, Dhaka, Bangladesh; 3 Rural Health Research Institute, Charles Sturt University, Orange, Australia

**Keywords:** Enterovirus 71 (EV71), Hand-foot-and-mouth disease, Neutralizing antibody, Seroprevalence, Children, Bangladesh

## Abstract

**Objectives:**

The world has witnessed an increase in non-polio enterovirus infections. This study assessed the seroprevalence of enterovirus 71 (EV71) neutralizing antibodies (NAbs) and explored associated risk factors among children and adolescents in Bangladesh.

**Methods:**

Between March 2019 and March 2020, a cross-sectional survey was conducted in one urban and one rural district of Bangladesh. A total of 1207 children aged 0-17 years were enrolled, including 607 from urban and 600 from rural areas. Serum samples were tested using a cytopathic effect-based microneutralization assay to measure EV71 NAb titers. Demographic and socio-epidemiological data were also collected through structured interviews.

**Results:**

Overall, 817 (67.7%) were seropositive for EV71, with 68.3% in urban and 31.7% in rural areas. Age-specific seroprevalence in urban vs rural settings was: <6 months, 96.0% vs 38.9%; 6-71 months, 90.3% vs 35.1%; 6-11 years, 92.7% vs 80.4%; 12-17 years, 100% vs 77.6%. In urban areas, having an indoor toilet was linked to lower seropositivity (*P* = 0.03). In rural areas, older age, lower parental education, and parental unemployment were significant factors.

**Conclusions:**

The high EV71 seroprevalence in Bangladeshi children indicates widespread exposure. Further research on non-polio enterovirus, including EV71, is needed to inform surveillance, prevention, and potential vaccine strategies.

## Highlights

• Reporting seroprevalence of enterovirus 71 neutralizing antibodies first time from Bangladesh.
• High seroprevalence of enterovirus 71 neutralizing antibodies among Bangladeshi children.
• Seropositivity correlated with demographic and socio-economic factors.
• These findings underscore gaps in research on enterovirus in Bangladesh.

## Introduction

Enteroviruses (EVs), a genus of the Picornaviridae family, comprise over 300 distinct serotypes [1,2]. Phylogenetic studies have identified 15 EV species, including polioviruses, non-polio enteroviruses, and rhinoviruses [3,4]. Seven of these species (EVs A to D and Rhinoviruses A to C) are known to cause human diseases [2,3]. EV 71 (EV71 or EVA71) is a single-stranded positive-sense RNA virus with a genome of approximately 7500 bases [5].

Globally, EV71 infection poses a serious public health risk [6,7]. In children under 7, EV71 typically causes enteroviral vesicular stomatitis hand-foot-and-mouth disease (HFMD), herpangina and neurological abnormalities [8]. While both EV71 and coxsackievirus 16 (CVA16) are predominant causative agents of HFMD, EV71 is considered the primary pathogen [9]. Serious health risks associated with HFMD include respiratory and cardiovascular disorders such as cardiopulmonary failure, fulminant neurogenic pulmonary oedema, as well as neurological disorders such as central nervous system abnormalities, aseptic meningitis, cerebella ataxia, acute brainstem encephalitis, poliomyelitis-like paralysis, and even death [10].

In Asia, HFMD has emerged as an infectious disease of heightened pathogenic potential, requiring the attention of the national and international medical community. Large epidemics of HFMD caused by CVA16 and EVA71 suggest that enterovirus A is the most common species in Asia [11]. By the 1990s, EV71 had become endemic in the Asia-Pacific region, triggering widespread outbreaks every 3 to 4 years. Countries such as Malaysia [12], Taiwan [13], and Singapore [14] have experienced recent epidemics. In the Asia-Pacific region, the mortality rate of EV71-associated HFMD has ranged from 0.5-19% [15]. Studies on the seroprevalence of EV71 in Singapore, Brazil, Taiwan and China [[16], [17], [18], [19], [20], [21], [22]] have shown wide variations in neutralizing antibody (Nab) seroprevalence by age, ranging from 20-60% [21,22]. While Bangladesh experiences HFMD, it may not be typically associated with a large-scale EV71 epidemic. Notably, Bangladesh has no national immunization policy for EV71, despite the availability of effective EV71 vaccines.

Cell-mediated immunity is the primary natural defense against enterovirus and EV71, while humoral immunity with NAbs is also critical for safeguarding [23]. Although sporadic incidences and outbreaks of HFMD are reported, there is currently no data on the prevalence of neutralizing antibodies against enteroviruses, including EV71, in Bangladesh. While seroprevalence does not directly indicate clinical disease prevalence, age-specific seroprevalence of EV71 antibodies in a vaccine-naïve population like Bangladesh can provide insights into the endemicity and risk of infection across different age groups. This study aimed to determine the seroprevalence of EV71 NAbs among children and identify risk factors influencing antibody levels in selected rural and urban populations of Bangladesh.

## Materials and methods

### Study design

**Figure 1:**
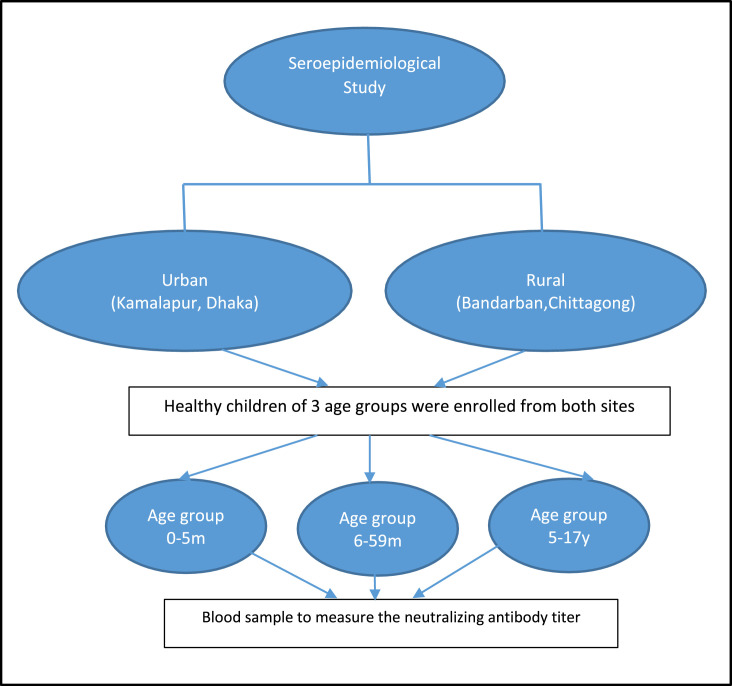
Cross-sectional study design. m, month; y, year.

A cross-sectional EV71 seroprevalence survey was conducted among children and adolescents in Bangladesh (Figure 1).

### Study sites

The study was conducted in two Health and Demographic Surveillance Systems (HDSS) of icddr,b: Kamalapur HDSS in metropolitan Dhaka and Bandarban Hill Tracks HDSS in a rural area. The Kamalapur HDSS had a population of approximately 300,000 residents, with 11% of the population under 5 years of age. Urban families living in this district had an average monthly income of Bangladeshi Taka 5000 (US$ 45). The average education for men over 18 was 4.5 years, and for women was 3.1 years. The Bandarban Hill Tracks HDSS had a population of 292,900 and is located in the southeast region of the country, known as one of the remotest and least populated districts of the country.

### Recruitment strategy

Before the study commenced, the research team visited HDSS households to compile a list of potential participants across three age categories. During these visits, team members sought written consent, conducted a preliminary eligibility assessment, and collected data for household conditions, hygiene practices, and other demographics. Parents were then advised to bring their children to the HDSS clinic for further evaluation. At the clinic, study doctors assessed each child’s eligibility, reviewed their medical history, and performed a routine physical examination, which included measuring vital signs. Each eligible participant was assigned a unique identification number.

### Eligibility criteria

Children meeting the following inclusion criteria were enrolled between March 12, 2019, and March 24, 2020: healthy individuals of either sex, aged 0-17 years, who provided signed informed consent, could adhere to study procedures, and were available for the entire study period. Exclusion criteria included fever (axillary temperature of higher than 38.1°C [100.5°F]); rash; use of antibiotics or antivirals within the past 2 weeks; corticosteroid treatment within the past 30 days, or receipt of blood transfusions or blood products, including immunoglobulins, in the past 4 weeks.

### Sample size calculation

In the absence of seroprevalence data on NAbs against EV71, we calculated the sample size based on seroprevalence data from other Southeast Asian countries [16,21,24,25]. We estimated a 50% seroprevalence in children aged 6-59 months. Using the formula, *n = Z^2^P(1- P)/d^2^*, we computed the sample size needed to estimate NAb prevalence for children <6 months, 6-59 months, and 5-17 years, with a 95% confidence level. Here, n is the sample size, *Z* is the statistic with a 95% confidence interval (CI), P is the predicted prevalence, and *d* is the precision [26]. Consequently, 1210 participants were enrolled.

### Serum sample collection

Three ml (or <2.5 ml for children ≤2 years of age) of venous blood were collected from 1207 participants following the standard clinical specimen collection protocol [27]. For serum separation, the blood samples were centrifuged at 3500 rpm for 5 minutes. The resulting serum samples were then stored at –80°C in ultra-low temperature conditions until use in the microneutralization assay. The serum samples were shipped to the National Polio and Measles-Rubella Laboratory (NPML) of the Institute of Public Health for microneutralization testing. After incubation, an antibody titer of 1:8 or higher was used to determine seropositivity [25,28].

### Microneutralization assay

A cytopathic effect-based microneutralization assay was performed using 96-well micro-culture plates, with human rhabdomyosarcoma cells cultured for the analysis. The NAb test for the C4 genotype of EV71 strain (523-071) was performed. From each stored serum sample, a 100 μl aliquot was inactivated at 56°C for 30 minutes, with intermittent shaking every 15 minutes. The serum was then two-fold serially diluted in Eagle’s Minimum Essential Medium from 1:8 to 1:1024. The diluted serum was mixed with the test virus solution at a concentration of 100 CCID50/0.05 ml and incubated at 37°C for 2 hours. After this, 100 μl of the mixture containing virus and serum was added to the rhabdomyosarcoma cell monolayer in the well and incubated for 5-6 days at 37°C. Each assay plate included a positive control with a known antibody titer, along with controls for cell culture and viral back-titration, to ensure accurate virus inoculum quantification at concentrations of 0.1, 1, 10, and 100 CCID50/0.05 ml. The test was repeated, and the presence of cytopathic effect (CPE) indicated viral infection in the inoculated cell monolayers. The antibody titer was defined as the reciprocal of the highest serum dilution that protected 50% of the inoculated cell monolayers from infection, that is, the serum dilution with a CPE score of <2+. A titer of 1:8 or higher was indicative of seropositivity [25,28].

### Statistical analysis

Statistical analyses were performed using Stata 15.1 (Stata Corporation, College Station, Texas). Descriptive statistics were used to assess the baseline characteristics of the study participants. Categorical and continuous data were presented as percentages, mean ± SD, or median. Univariate logistic regression was employed to examine the relationship between independent factors and EV71 seropositivity, with a *P*-value of <0.05 considered statistically significant. Odds ratio (OR) and 95% CI were calculated. Multivariate logistic regression analysis was performed for the important risk factors (age, location of toilet, monthly income, parental education and occupation, and personal hygiene), and adjusted OR (aOR) and 95% CI were reported. We measured antibody titers across age groups after log transformation to geometric mean titers (GMTs).

## Results

### Enrolment

**Figure 2:**
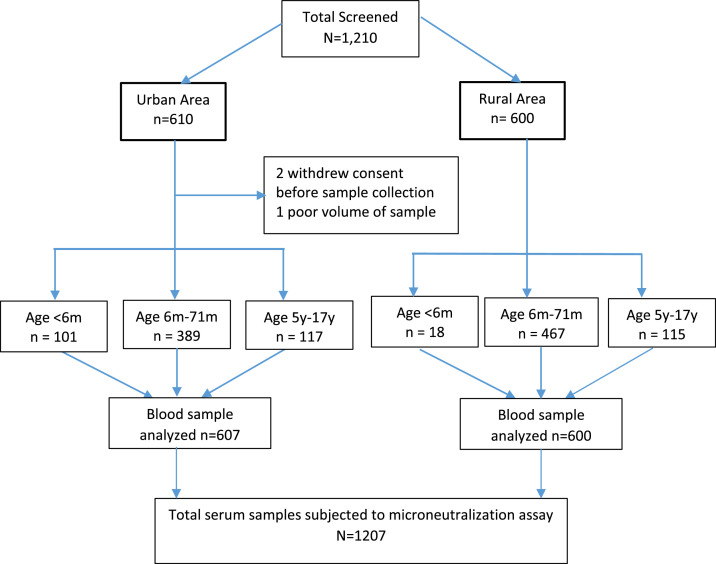
Participant enrolment and sample collection. m, month; y, year.

A total of 1210 children, 0 to 17 years of age, were enrolled between March 12, 2019, and March 24, 2020. Of these, 610 were from the urban areas and 600 were from the rural localities. Two participants withdrew before sample collection, and one sample was excluded due to poor quality, leaving 1207 serum samples (607 urban and 600 rural) for testing. Overall, 817 (67.7 %) participants were EV71 seropositive, with 558 (68.3%) from urban areas and 259 (31.7%) from rural areas. The flow of enrolment is shown in Figure 2.

### Seroprevalence

#### Urban prevalence

**Figure 3:**
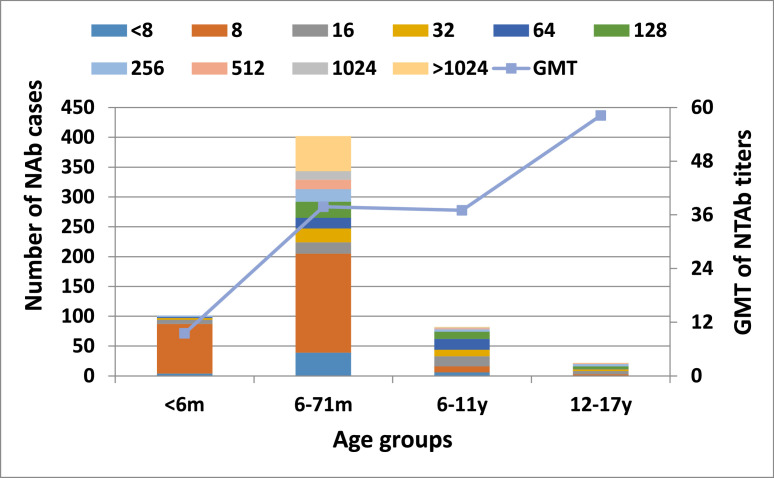
Antibody titer distribution and geometric mean titer in the urban population. m, month; y, year.

In the 607 urban samples, the overall EV71 seroprevalence rate was 91.9% (n = 558), with a GMT of 30.4. The age-specific seroprevalence rates were 96.0% for children under 6 months, 90.3% for those aged 6-71 months, 92.7% for those aged 6-11 years, and 100.0% for those aged 11-17 years. The highest GMT (58.2) was observed in the 11-17 year age group (Figure 3; Table 1). The corresponding GMT values for the younger age groups were 9.8, 48.1, and 44, respectively (Table 1). Multivariate analysis revealed that only the presence of a toilet within the household was associated with a reduced risk of EV71 seropositivity (aOR: 0.51; 95% CI: 0.28-0.92; *P* = 0.03).

**Table 1:** Summary of antibody titer distribution and geometric mean titer in the urban and rural sample.

Location	Urban				Rural			
Age	<6 m	6-71 m	6-11 y	12-17 y	<6 m	6-71 m	6-11 y	12-17 y
Number of participants	101	402	82	22	18	473	51	58
Seropositive cases n (%)	97 (96.0)	363 (90.3)	76 (92.7	22 (100.0)	7 (38.9)	166 (35.1).4)	41 (80.4)	45 (77.6)
Geometric mean titer	9.8	48.1	44.0	58.2	5.2	13.6	42.0	19.4

m, month; y, year.

#### Rural prevalence

**Figure 4:**
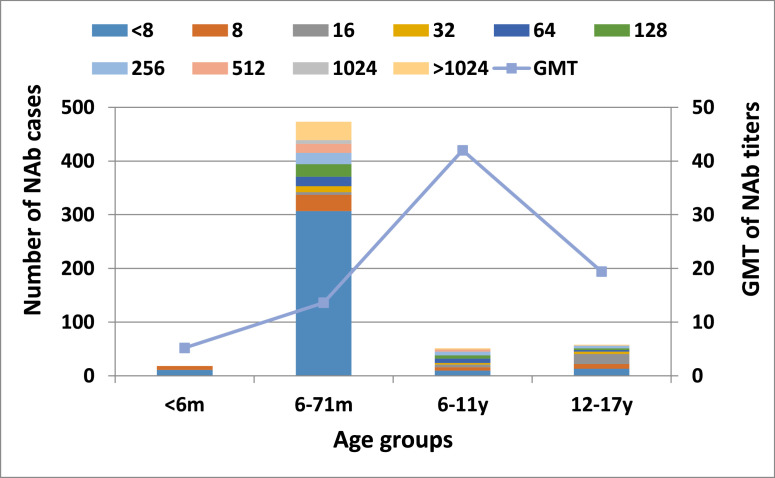
Antibody titer distribution and geometric mean titer in the rural populations. m, month; y, year.

In rural areas, 259 of 600 participants (43.2%) were seropositive for EV71, with a corresponding GMT of 15.0. The age-specific seroprevalence rates were 38.9% for children under 6 months, 35.1% for those aged 6-71 months, 80.4% for those aged 6-11 years, and 77.6% for those aged 12-17 years. The highest GMT (42.0) was observed in the 6-11 years age group (Figure 4; Table 1), with corresponding values of 5.2, 13.6, and 19.4 for the other age groups (Table 1). Children aged 6-11 years were approximately six times more likely to be seropositive compared to those aged 0-5 months (aOR: 5.68; 95% CI: 1.63-19.80; *P* = 0.01). Similarly, children aged 12-17 years had a five-fold higher seropositivity rate (aOR 4.77; 95% CI 1.40-16.19; *P* = 0.01). Rural children of mothers with lower educational status (aOR: 1.68; 95% CI: 1.19-2.39; *P* = 0.003), and illiterate fathers (aOR: 4.27; 95% CI: 1.01-18.14; *P* = 0.049) had an increased risk of EV71 seropositivity. Counterintuitively, univariate analysis revealed that rural children greater than 4 years of age, with better personal hygiene (OR 3.13; 95% CI 1.64-5.95; *P* = 0.001), and from higher-income households (OR 1.92; 95% CI 1.33-2.77; *P* <0.001), or whose mothers worked outside the home (OR 1.96; 95% CI 1.39-2.77; *P* <0.001) were more likely to be seropositive. In multivariate analysis, seropositivity could not be correlated with higher-income households (aOR: 1.21; 95% CI 1.78-1.89; *P* = 0.40), or mother’s working status (aOR: 1.08; 95% CI: 0.68-1.70; *P* = 0.75) Conversely, the father’s unemployment status was negatively correlated with seropositivity (aOR: 0.59; 95% CI: 0.38-0.92; *P* = 0.02).

### Neutralizing antibody level

NAb titers were categorized into four groups: 1:8 (negative), 1:8–1:64 (low), 1:128 (medium), and 1:256 (high) [29]. In the urban population, 27% of children aged 6-71 months and 12-17 years had a high NAb titer (1:256), followed by the 6–11 years age group (9.8%). The most common medium NAb titer (1:128) was found in individuals aged 12-17 years (18.2%). The lowest titer levels (1:8–1:64) were seen in 94.1% of children under 6 months. In rural areas, 25.5% of children aged 6-11 years had high NAb titers (1:256), followed by 16.7% of children aged 6-71 months. The most common medium titer (1:128) was observed in 11.8% of children aged 6-11 years, while 60.3% of the 12-17-year age group had lower titers (1:8-1:64) (Table 2).

**Table 2:** Enterovirus 71 neutralizing antibody titer distribution by age group in the urban and rural sample.

Distribution of titer	Percentage (%) of titer level in participants of different age groups							
	Urban				Rural			
	<6 m	6-71 m	6-11 y	12-17 y	<6 m	6-71 m	6-11 y	12-17 y
<1:8 (negative titer level)	4.0	9.7	7.3	0.0	61.1	64.9	19.6	22.4
1:8-1:64 (low-level titer)	94.1	56.2	68.3	54.6	38.9	13.5	43.1	60.3
1:128 (medium titer level)	0.0	6.7	14.6	18.2	0.0	4.9	11.8	5,2
≥1:256 (high titer level)	2.0	27.4	9.8	27.3	0.0	16.7	25.5	12.1

m, month; y, year.

## Discussion

This was the first study in Bangladesh to determine the seroprevalence and risk factors for EV71 NAb seropositivity among children and adolescents aged 0-17 years. The findings revealed that children older than 71 months exhibited a higher seroprevalence rate and GMT of EV71 NAbs, suggesting a greater exposure to the virus in this age group. In addition, the study demonstrated that children in selected rural areas under 6 months of age were the most susceptible to EV71 infection, followed by those aged 6-71 months, with seropositivity rates of 35.1% and 38.9%, respectively. No significant gender differences in seropositivity rates were observed, which is consistent with findings from other studies [18,30,31]. The seroprevalence rate in urban areas closely aligned with the overall seroprevalence of EV71 NAbs (88.8%) among children aged 2–15 years from Cambodia [30]. However, these findings contrast with a study from Taiwan, which reported higher post-epidemic EV71 NAb seropositivity in preschool-aged children from rural areas compared to urban settings [29].

High seroprevalence rates across all age groups of urban children suggest widespread exposure to the EV71 viruses. This can be due to factors such as high transmission rates in densely populated communities, particularly in the urban slum areas of Dhaka. Poor sanitation and waste management in these areas can further facilitate the spread of EV1 infection. Additional investigation is needed to better understand the underlying drivers of these infection dynamics.

The high frequency of EV71 seropositivity (96%) among urban children under 6 months of age in this study may be attributed to the presence of maternal antibodies. This finding is consistent with a meta-analysis of 42 global studies, which found that an average of 78% of neonates were seropositive for EV71 due to transplacental maternal antibodies. In our study, the higher seropositivity observed in children under 6 months supports this finding. The meta-analysis also suggested that such transplacental maternal antibody wanes by approximately 5 months [24]. Few studies have reported seropositivity rates (89%) as high as those found in our study [30].

The larger difference between the seropositivity rate in the <6 months age group in urban and rural areas may reflect reduced exposure of rural mothers to EV71. Higher population density and poor sanitation in urban slum areas compared to rural areas may be a contributing factor. Another possible explanation is that in rural areas, we were unable to reach the intended sample size for children younger than 6 months and compensated by recruiting an additional 157 children aged 6-71 months.

The seroprevalence rate of 38.9% among rural children under 6 months of age closely mirrors a study from Thailand, which reported a seroprevalence of 30-37% in the same age group [25]. Additionally, the Thailand study found a seroprevalence rate of approximately 75-90% among children 5 to 17 years, similar to the rates observed in older rural children in our study [25]. Our results revealed that rural children older than 6 years had up to an 80% increase in seroprevalence, consistent with other studies [32]. The low seropositivity among rural children under 6 years of age and gradual increase with age align with the findings from studies conducted in Taiwan and mainland China, where EV71 seroprevalence also increased with age [18].

The GMT of EV71 antibody peaked among urban children under 6 months of age and increased to 48 in children aged 6-71 months. The GMT reached its highest level in adolescents aged 12-17 years, with a slight decline observed in the 6-11-year age group. These findings, however, differ from those of a meta-analysis of 42 studies worldwide, which indicated that the GMT of EV71 antibodies decreases after peaking at 5 years of age [24].

The location of the toilet outside the household was one of the major risk factors linked to the increased seropositivity of EV71 in urban children. The presence of a toilet inside the household may contribute to maintaining good hygiene and minimize the potential for pathogen transmission. Higher seropositivity among rural younger children with better personal hygiene may indicate improper or compromised personal hygiene practices that might negatively impact infection control. Children whose mothers worked outside the home might be cared for by persons with lower levels of educational attainment and hygiene awareness, which contributed to higher rates of seropositivity. However, further investigation into the intricate relationship of these risk factors is needed.

This study had several strengths. First, it used a community-based design and random sampling, the most reliable approach for obtaining a representative sample in a seroprevalence survey. Second, antibody titers were measured using the microneutralization assay at the World Health Organization-qualified NPML at the Institute of Public Health, Bangladesh, ensuring reliable results. However, there were also limitations. This was a cross-sectional study, which provides only a snapshot in time. EV71 immunity is dynamic and may vary with disease severity, natural waning of antibody, reinfection, vaccination, immune maturity, and nutritional and health status. A longitudinal design would be better suited to assess the duration of seropositivity. We also did not determine the proportion of infections that cause clinically significant illness, which would have allowed correlation between NAb titers and disease severity. In addition, our chosen antibody titer threshold of 8, together with the lack of standardized neutralization assays for EV71, could affect comparability with other studies. Cross reactivity with other concurrently circulating enteroviruses could also have led to false-positive results, particularly at low antibody titers.

Seroprevalence studies in other countries, such as China, have provided valuable insights into age-specific susceptibility, infection dynamics, and the potential impact of vaccination, thereby influencing public health strategies and vaccine polices, including determining the optimal age group for immunization [24]. Similar well-designed studies are essential in Bangladesh to improve understanding of EV71 epidemiology and inform effective presentation measures.

## Conclusion

Bangladesh is particularly vulnerable to enterovirus infection because of its tropical climate. Anecdotal evidence suggests that HFMD outbreaks commenced in Dhaka, the capital, in September 2022, with most cases occurring in children under 10 years of age. Due to the absence of reliable diagnostic methods and the fact that HFMD is a contagious but reportable illness, it is difficult to determine its true prevalence in Bangladesh or to identify its primary causative agents.

Many cases may go unreported because HFMD is often mild and self-limiting. However, if left untreated, it can cause severe impairment or even death in children. In our study, a substantial prevalence of EV71 neutralizing antibodies was detected among selected children and adolescents in both urban and rural areas. These findings prioritize the need for further research to assess the burden of EV71 and other non-polio enteroviruses in the general population. Further longitudinal seroprevalence studies will be important for understanding serotype dynamics and assessing the effectiveness of new vaccines. If our results are validated, the inclusion of the EV71 SINOVAC Inlive® vaccine in the national immunization program for children aged 6 months to 3 years could be considered.

## Declaration of competing interest

The authors have no competing interests to declare.

## Appendix Supplementary materials

Image, application 1
