# Supplementary material for: Enterovirus 71 seropositivity among children and adolescents in Bangladesh
Source: IJID Reg. 2025 Aug 23;17:100739. doi: 10.1016/j.ijregi.2025.100739 (PMC12746088; doi:10.1016/j.ijregi.2025.100739)
Supplement: Supplementary file 1 [file mmc1.docx]

**Appendix**

Table 3. Key characteristics of the urban participants and their EV71 seropositivity results.

|  | **Participants,**  **N (%)** | | OR (95% CI) | P-Value | Adjusted OR (95% CI) | P-Value |
| --- | --- | --- | --- | --- | --- | --- |
|  | **Urban (607)^*^** | |  |  |  |  |
| **Characteristic** | **Total** | **Seropositive**  **For EV71** |  |  |  |  |
| **Total Participant** | 607 | 558 (91.9) |  |  |  |  |
| **Individual factors** |  |  |  |  |  |  |
| **Religion** | | |  |  |  |  |
| Muslim | 564 | 520 (92.2) | Reference |  |  |  |
| Hindu/Buddhist | 43 | 38 (88.4) | 0.64 (0.24 - 1.72) | 0.38 |  |  |
| **Age-group** |  |  |  |  |  |  |
| 0m-5 months | 101 | 97 (96.0) | Reference |  | Reference |  |
| 6m- 71 months | 402 | 363 (90.3) | 0.38 (0.13 - 1.10) | 0.08 | 0.38 (0.13 - 1.08) | 0.07 |
| 6-11 years | 82 | 76 (92.7) | 0.52 (0.14 - 1.92) | 0.36 | 0.53 (0.14 - 1.96) | 0.34 |
| 12-17 years | 22 | 22 (100.0) | ----- |  | ----- |  |
| **Sex** | | |  |  |  |  |
| Male | 281 | 261 (92.9) | Reference |  |  |  |
| Female | 326 | 297 (91.1) | 0.79 (0.43 - 1.42) | 0.42 |  |  |
| **BMI percentile (0m-5y) n=503** | | |  |  |  |  |
| <85^th^ percentile | 428 | 392 (91.6) | Reference |  |  |  |
| ≥85^th^ percentile | 75 | 68 (90.7) | 0.89 (0.38 - 2.09) | 0.79 |  |  |
| **Education, (6y-17y) n=104** | | |  |  |  |  |
| < 5 years | 85 | 79 (92.9) | Reference |  |  |  |
| ≥ 5 years | 19 | 19 (100.0) | ----- |  |  |  |
| **Occupation, (6y-17y) n=104** | | |  |  |  |  |
| Student | 97 | 91 (93.8) | Reference |  |  |  |
| Other | 7 | 7 (100.0) | ----- |  |  |  |
| **History of breastfeeding, (<6m) n=101** | | |  |  |  |  |
| Exclusive | 61 | 60 (98.4) | Reference |  |  |  |
| Non-Exclusive | 40 | 37 (92.5) | 0.21 (0.02 - 2.05) | 0.18 |  |  |
| **Duration of breastfeeding, (0m -59m) n=490** | | |  |  |  |  |
| ≥6 months | 348 | 315 (90.5) | Reference |  |  |  |
| <6 months/occasional/never | 142 | 134 (94.4) | 1.75 (0.79 - 3.90) | 0.18 |  |  |
| **Personal Hygiene Practice (**For 0-48m) **n=427** | | |  |  |  |  |
| Hygiene index **≤5** | 48 | 44 (91.7) | Reference |  |  |  |
| Hygiene index >5 | 379 | 346 (91.3) | 0.95 (0.32 - 2.82) | 0.93 |  |  |
| **Personal Hygiene Practice (**For >48m)) **n=180** | | |  |  |  |  |
| Hygiene index **≤6** | 16 | 16 (100.0) | Reference |  |  |  |
| Hygiene index >6 | 164 | 152 (92.7) | ------- |  |  |  |
| **History of School attendance (≥6 years old), n=104** | | |  |  |  |  |
| Regular | 92 | 86 (93.5) | Reference |  |  |  |
| Irregular | 12 | 12 (100.0) | ------- |  |  |  |
| **History of contact with Person suspected to have HFMD infection, n=607** | | | | |  |  |
| Yes | 5 | 5 (100.0) | Reference |  |  |  |
| No/ Don't Know | 602 | 553 (91.9) | ------- |  |  |  |
| **Household factors** |  |  |  |  |  |  |
| **Number of household members** | | |  |  |  |  |
| **≤5** | 414 | 377 (91.1) | Reference |  |  |  |
| **>5** | 193 | 181 (93.8) | 1.48 (0.75 - 2.91) | 0.26 |  |  |
| **Monthly income** | | |  |  |  |  |
| ≤20000 | 436 | 403 (92.4) | Reference |  |  |  |
| >20000 | 171 | 155 (90.6) | 0.79 (0.42 - 1.48) | 0.47 |  |  |
| **Mother’s education** | | |  |  |  |  |
| ≥ 8 years | 179 | 168 (93.9) | Reference |  |  |  |
| < 8 years | 334 | 300 (90.4) | 0.62 (0.30 - 1.26) | 0.18 |  |  |
| Never attended/ Unknown/NA | 94 | 88 (93.6) | 0.96 (0.34 - 2.68) | 0.94 |  |  |
| **Mother’s occupation** | | |  |  |  |  |
| Unemployed | 516 | 471 (91.3) | Reference |  |  |  |
| Employed | 91 | 87 (95.6) | 2.08 (0.73 - 5.93) | 0.17 |  |  |
| **Father’s education** | | |  |  |  |  |
| ≥ 8 years | 212 | 196 (92.4) | Reference |  |  |  |
| < 8 years | 243 | 215 (88.5) | 0.63 (0.33 - 1.19) | 0.16 |  |  |
| Never attended/ | 152 | 147 (96.7) | 2.40 (0.86 - 6.70) | 0.10 |  |  |
| **Father’s Occupation** | | |  |  |  |  |
| Day Labourer | 313 | 288 (92.0) | Reference |  |  |  |
| Employed in private or public sector | 95 | 83 (87.4) | 0.60 (0.29 - 1.25) | 0.17 |  |  |
| Business | 142 | 132 (93.0) | 1.15 (0.53 - 2.45) | 0.73 |  |  |
| Unemployed | 57 | 55 (96.0) | 2.38 (0.55 - 10.37) | 0.25 |  |  |
| **Water source** | | |  |  |  |  |
| Supply/Tubewell | 466 | 427 (91.6) | Reference |  |  |  |
| Natural body and other | 141 | 131 (92.9) | 1.20 (0.58 - 2.46) | 0.63 |  |  |
| **Drinking water quality** | | |  |  |  |  |
| Unboiled/Other | 342 | 320 (93.6) | Reference |  |  |  |
| Boiled/Purified | 265 | 238 (89.8) | 0.61 (0.34 - 1.09) | 0.10 |  |  |
| **Toilet type** | | |  |  |  |  |
| Pakka/Pit/Slab | 606 | 557 (91.9) | Reference |  |  |  |
| Kaccha/other | 1 | 1 (100.0) | ------- |  |  |  |
| **Location of Toilet** | | |  |  |  |  |
| Outdoor | 375 | 352 (93.9) | Reference |  | Reference |  |
| Indoor | 232 | 206 (88.8) | 0.52 (0.29 - 0.93) | 0.03 | 0.51 (0.28 - 0.92) | 0.03 |

Table 4. Key Characteristics of rural participants and their EV71 seropositivity results.

|  | **Participants,**  **N (%)** | | OR (95% CI) | P-Value | Adjusted OR (95% CI) | P-Value |
| --- | --- | --- | --- | --- | --- | --- |
|  | **Rural (600)^*^** | |  |  |  |  |
| **Characteristic** | **Total** | **Seropositive**  **For EV71** |  |  |  |  |
| **Total Participant** | 600 | 259 (43.2) |  |  |  |  |
| **Individual factors** |  |  |  |  |  |  |
| **Religion (N=600)** | | |  |  |  |  |
| Muslim | 189 | 85 (45.0) | Reference |  |  |  |
| Hindu/ Christian/ Buddhist | 411 | 174 (42.3) | 0.90 (0.63 - 1.27) | 0.54 |  |  |
| Ethnicity **(N=600)** |  |  |  |  |  |  |
| Non-tribal | 226 | 103 (45.6) | Reference |  |  |  |
| Tribal | 374 | 156 (41.7) | 0.86 (0.61 - 1.19) | 0.36 |  |  |
| **Age-group (N=600)** |  |  |  |  |  |  |
| 0-5 months | 18 | 7 (38.9) | Reference |  | Reference |  |
| 6- 71 months | 473 | 166(35.0) | 0.85(0.32 - 2.23) | 0.74 | 0.82(0.31 - 2.19) | 0.69 |
| 6-11 years | 51 | 41 (80.4) | 6.44 (1.99 – 20.80) | 0.002 | 5.68 (1.63 – 19.80) | 0.01 |
| 12y-17 years | 58 | 45 (77.6) | 5.44 (1.76 – 16.85) | 0.003 | 4.77 (1.40 – 16.19) | 0.01 |
| **Sex** (N=600) | | |  |  |  |  |
| Male | 306 | 141 (46.1) | Reference |  |  |  |
| Female | 294 | 118 (40.1) | 0.79 (0.57 - 1.09) | 0.142 |  |  |
| **BMI percentile (0m-5y) n= 489** | | |  |  |  |  |
| <85^th^ percentile | 448 | 162 (36.2) | Reference |  |  |  |
| ≥85^th^ percentile | 41 | 10 (24.4) | 0.57 (0.27 - 1.91) | 0.78 |  |  |
| **Education, n=109** | | |  |  |  |  |
| < 5 years | 64 | 50 (78.1) | Reference |  |  |  |
| ≥ 5 years | 45 | 82.2 (79.8) | 1.30 (0.49 - 3.41) | 0.60 |  |  |
| **Occupation, n=109** | | |  |  |  |  |
| Student | 101 | 83 (82.2) | Reference |  |  |  |
| Other | 8 | 4 (50.0) | 0.22 (0.05 - 0.95 | 0.04 |  |  |
| **History of breastfeeding, (N=18)** | | |  |  |  |  |
| Exclusive | 6 | 4 (66.7) | Reference |  |  |  |
| Non-Exclusive | 12 | 3 (25.0) | 0.17 (0.02 - 1.42) | 0.101 |  |  |
| **Duration of breastfeeding (0m -59m), (N=485)** | | |  |  |  |  |
| ≥6 months | 4 | 0 (0.00) | Reference |  |  |  |
| <6 months/occasional/never | 484 | 168 (34.9) | ----- |  |  |  |
| **Personal Hygiene Practice (**For 0-48m) (N=411) | | |  |  |  |  |
| Hygiene index **≤5** | 191 | 59 (30.9) | Reference |  |  |  |
| Hygiene index >5 | 220 | 70 (31.8) | 1.04 (0.69 - 1.59) | 0.84 |  |  |
| **Personal Hygiene Practice (**For >48m)) (N=189) | | |  |  |  |  |
| Hygiene index **≤6** | 65 | 34 (52.3) | Reference |  |  |  |
| Hygiene index >6 | 124 | 96 (77.4) | 3.13 (1.64 - 5.95) | 0.001 |  |  |
| **History of School attendance (≥6 years old), n=109** | | |  |  |  |  |
| Regular | 100 | 82 (82.0) | Reference |  |  |  |
| Irregular/Not applicable | 9 | 5 (55.6) | 0.27 (0.07 - 1.12) | 0.07 |  |  |
| **History of contact with Person suspected to have HFMD infection (N=600)** | | | | |  |  |
| Yes | 0 | 0 (0.0) | Reference |  |  |  |
| No/ Don't Know | 600 | 259 (43.2) | ------- |  |  |  |
| **Household factors** |  |  |  |  |  |  |
| **Number of household members (N=600)** | | |  |  |  |  |
| **≤5** | 403 | 173 (42.9) | Reference |  |  |  |
| **>5** | 197 | 86 (43.7) | 1.03 (0.73 - 1.45) | 0.87 |  |  |
| **Monthly income (N=600)** | | |  |  |  |  |
| ≤20000 | 440 | 171 (38.9) | Reference |  | Reference |  |
| >20000 | 160 | 88 (55.0) | 1.92 (1.33 - 2.77) | <0.001 | 1.21 (0.78 - 1.89) | 0.40 |
| **Mother’s education (N=600)** | | |  |  |  |  |
| ≥ 8 years | 207 | 72 (34.8) | Reference |  |  |  |
| < 8 years | 391 | 185 (47.3) | 1.68 (1.19 - 2.39) | 0.003 |  |  |
| Never attended | 2 | 2 (100.0) | ----- |  |  |  |
| **Mother’s occupation (N=600)** | | |  |  |  |  |
| Unemployed | 405 | 153 (37.8) | Reference |  | Reference |  |
| Employed/ | 195 | 106 (54.4) | 1.96 (1.39 - 2.77) | <0.001 | 1.08 (0.68 - 1.70) | 0.75 |
| **Father’s education (N=600)** | | |  |  |  |  |
| ≥ 8 years | 212 | 81 (38.2) | Reference |  | Reference |  |
| < 8 years | 375 | 168 (44.8) | 1.31 (0.93 - 1.85) | 0.12 | 1.17(0.79 - 1.72) | 0.44 |
| Never attended | 13 | 10 (76.9) | 5.39 (1.44 - 20.17) | 0.01 | 4.27(1.01 – 18.14) | 0.049 |
| **Father’s Occupation (N=600)** | | |  |  |  |  |
| Day Labourer | 269 | 121 (45.0) | Reference |  | Reference |  |
| Employed in private or public sector | 41 | 10 (24.4) | 0.40 (0.19 - 0.84) | 0.01 | 0.48 (0.21 - 1.07) | 0.07 |
| Business | 94 | 45 (47.9) | 1.12 (0.70 - 1.80) | 0.63 | 0.92 (0.55 - 1.55) | 0.76 |
| Not employed | 196 | 83 (42.3) | 0.90 (0.62 - 1.30) | 0.57 | 0.59 (0.38 - 0.92) | 0.02 |
| **Water source (N=600)** | | |  |  |  |  |
| Supply/Tubewell | 561 | 247 (44.0) | Reference |  |  |  |
| Natural body and other | 39 | 12 (30.8) | 0.57 (0.28 - 1.14) | 0.11 |  |  |
| **Drinking water quality (N=600)** | | |  |  |  |  |
| Unboiled/ Other | 570 | 249 (43.7) | Reference |  |  |  |
| Boiled/Purified | 30 | 10 (33.3) | 0.64 (0.30 - 1.40) | 0.27 |  |  |
| **Toilet type (N=600)** | | |  |  |  |  |
| Pakka/Pit/Slab | 511 | 226 (44.2) | Reference |  |  |  |
| Kaccha/other | 89 | 33 (37.1) | 0.74 (0.47 - 1.18) | 0.21 |  |  |
| **Location of Toilet (N=600)** | | |  |  |  |  |
| Outdoor | 571 | 248 (43.4) | Reference |  |  |  |
| Indoor | 29 | 11 (37.9) | 0.80 (0.37 - 1.71) | 0.56 |  |  |
